# Effects of Fiber Angle on the Tensile Properties of Partially Delignified and Densified Wood

**DOI:** 10.3390/ma13235405

**Published:** 2020-11-27

**Authors:** Matthias Jakob, Jakob Gaugeler, Wolfgang Gindl-Altmutter

**Affiliations:** Department of Material Sciences and Process Engineering, Institute of Wood Technology and Renewable Materials, BOKU—University of Natural Resources and Life Sciences, Vienna, Konrad Lorenz-Strasse 24, 3430 Tulln, Austria; hnr@boku.ac.at (J.G.); wolfgang.gindl-altmutter@boku.ac.at (W.G.-A.)

**Keywords:** anisotropy, chemical treatment, delignification, densification, mechanical performance, wood compression, wood modification

## Abstract

Partial delignification and densification provide a pathway to significant improvement in the mechanical performance of wood. In order to elucidate potential effects of this treatment on the mechanical anisotropy of wood, partially delignified and densified spruce wood veneers were characterized at varying degrees of off-axis alignment. While the tensile strength and the modulus of elasticity (MOE) were clearly improved in parallel to the axis of wood fibers, this improvement quickly leveled off at misalignment angles ≥30°. For transverse tensile strength, the performance of alkaline-treated and densified wood was even inferior to that of untreated wood. Microscopic examination revealed the presence of microscopic cracks in treated wood, which are assumed to be responsible for this observation. It is concluded that impaired transverse tensile properties are a weakness of partially delignified and densified wood and should be considered when a potential usage in load-bearing applications is intended.

## 1. Introduction

Cellulose-based materials such as wood and natural fibers are experiencing a renewal of interest after a reduction of their use in parallel to the rise of steel and alloy steels during the 19th century, and synthetic polymers during the 20th century. This renaissance is largely due to growing awareness towards attributes such as biodegradability, eco-friendliness, and renewability [1,2,3]. Wood is a hierarchically structured natural polymer composite with cellular architecture. Roughly, it consists of 50% cellulose, 25% hemicelluloses, and 25% aromatic lignin. Cellulose is the main structural polymer in plant cell walls and consists of ß 1–4 linked glucose units, which form elongated molecules with several thousand repeat units. Hydrogen bonding enables the formation of intra- and intermolecular linkages and, consequently, crystalline structures with high mechanical strength of 1.6 to 6.6 GPa [4,5] and stiffness of 140 to 150 GPa [6,7,8]. Cellulose molecules assemble into nanofibrillar structures, which are arranged in a steep spiral in the largest part of the layered wood cell wall [9]. The resulting cell wall structure is similar to a unidirectional fiber-reinforced composite, with pronounced mechanical anisotropy caused by the different mechanical characteristics of fibers (cellulose nanofibrils) and matrix (hemicelluloses and lignin) [10].

Small, defect-free wood specimens of various species exhibit a density between 0.13 and 1.23 g cm^−3^ [11]. In parallel, their tensile strength and modulus of elasticity (MOE) lie between 60 and 220 MPa and between 6 and 22 GPa, respectively [11]. In comparison to other materials, this results in good mechanical performance at comparably low density, enabling slender and wide-spanning structures [12]. The strong correlation between wood density and mechanical performance suggests densification as a method for performance improvement. Elevated temperature and humidity soften wood [13,14], enabling densification close to the maximum possible value of 1.5 g cm^−3^, which is the density of the bulk wood cell wall [15]. A partial removal of cell wall polymers, particularly rigid lignin, also facilitates compression transversally to the fiber direction and minimizes cell wall fractures. It is an additional advantage of this procedure that, on relative terms, the content of mechanically strong cellulose is increased at the expense of less strong hemicelluloses and lignin [16]. By combining delignification with densification, impressive improvements to values of 20–50 GPa for the MOE and 270–550 MPa for tensile strength were achieved [17,18,19]. Notably, these studies focused on mechanical performance improvement in the direction parallel to the wood fibers.

By applying a partial delignification step followed by densification, the bending strength and the MOE of spruce plywood samples could be increased by the factors of 2.4 and 3.5, respectively, as shown in a previous study [18]. Contrary to that, interlaminar shear strength did not match the improvement after partial delignification and densification and increased by a factor of 1.4–1.6 only. Since the fracture pattern showed a dominance of wood failure, the authors assumed that a decrease in interfibrillar adhesion across the fiber direction in partially delignified and densified wood was the reason for the reduced improvement. Therefore, performance across the fiber direction should also be considered when wood composites are made with delignified and densified wood.

The objectives of this research were (a) to demonstrate the mechanical anisotropy of partially delignified and densified wood tested in tension, (b) to compare the results with regard to the anisotropy of untreated wood, and (c) to find explanations for potential differences.

## 2. Materials and Methods

**Figure 1:**
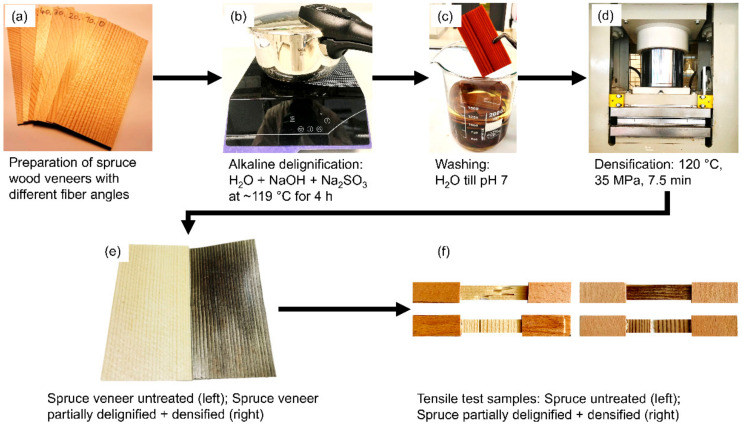
Key treatment steps in the preparation of partially delignified and densified spruce wood veneers.

Figure 1 gives an overview of all essential procedures applied in the present study. Sliced spruce wood veneers with even texture, an oven-dry density of ~0.36 g cm^−3^, and dimensions of 100 × 50 × 1.47 mm^3^ (axial × radial × tangential) were used as raw material and also served as reference samples. As shown in Figure 1a, specimens were cut with their longest edges in parallel to the direction of wood fibers, at an angle of 10° to the fiber axis, at an angle of 20°, and so forth up to an angle of 90°.

For partial delignification, veneers (n = 8 per fiber angle) were placed into a desiccator and were subsequently impregnated with deionized water (DW). To accelerate this process, a vacuum was applied by means of a vacuum pump. The veneers were then left under a vacuum at room temperature overnight until they were completely water-saturated. Afterward, 20 veneers (~50 g) were placed into a household pressure cooker (Sicomatic classic, WMF Group GmbH, Geislingen/Steige, Germany), shown in Figure 1b, containing an aquatic solution (1 L), mixed of 2.5 mol L^−1^ sodium hydroxide (NaOH) (Carl Roth GmbH + Co. KG, Karlsruhe, Germany) and 0.4 mol L^−1^ sodium sulfite (Na_2_SO_3_) (Carl Roth GmbH + Co. KG, Karlsruhe, Germany), as described by Song and coauthors [19]. Chemical treatment took place at ~119 °C (corresponding to a pressure of ~0.19 MPa) for 4 h. Subsequently, the treated veneers were repeatedly washed in DW until the washing water reached a stable pH of ~7 (Figure 1c). The washed and fully water-saturated veneers were then densified in the tangential direction in a hot press (Langzauner Gesellschaft m.B.H., Lambrecht, Austria), shown in Figure 1d, operated at a temperature of 120 °C, and a maximum pressure of 35 MPa was built-up stepwise during 7.5 min. After cooling under pressure to 60 °C, the specimens were left to equilibrate in a climate chamber operated at 20 °C and 65% humidity for at least two weeks.

The mass loss of each alkaline-treated and densified veneer (n = 8 per fiber angle) was calculated as

(1) $$ML\left(\%\right)=\frac{\left(m_{un}-m_{tr}\right)}{m_{un}}\times 100$$

where *ML* is the mass loss of the alkaline-treated and densified veneer, $m_{un}$ is the mass of the veneer before treatment, and $m_{tr}$ is the mass of the veneer after treatment. Afterward, the average mass loss was calculated.

The Klason lignin content was determined according to Nicholson and coauthors [20], without prior Soxhlet extraction by a 2:1 (*v*/*v*) mixture of toluene/ethanol, on untreated (n = 1) and uncompressed alkaline-treated (n = 1) specimens. Based on these results, and under the assumption that nearly no cellulose degradation occurred, an estimation of the new chemical composition of alkaline-treated specimens was calculated using the following equations [18]:

(2) $$ML_{kl}\left(\%\right)=KL_{un}-\left(\left(100-ML\right)\times \frac{KL_{tr}}{100}\right)$$

where $ML_{kl}$ is the mass loss due to Klason lignin delignification and $KL_{un}$ and $KL_{tr}$ are the Klason lignin contents of the untreated and uncompressed alkaline-treated specimens, respectively;

(3) $$ML_{h}\left(\%\right)=ML-ML_{kl}$$

where $ML_{h}$ is the mass loss due to hemicellulose degradation;

(4) $$H_{tr}\left(\%\right)=\frac{\left(H_{un}-ML_{h}\right)}{\left(100-ML\right)}\times 100$$

where $H_{tr}$ is the new hemicellulose content of the uncompressed alkaline-treated specimens and $H_{un}$ is the hemicellulose content of European spruce (31%) based on data from Fengel and Wegener [21]:

(5) $$C_{tr}\left(\%\right)=100-KL_{tr}-H_{tr}$$

where $C_{tr}$ is the new cellulose content of the uncompressed alkaline-treated specimens.

For mechanical testing, strips (n = 7 for reference samples (0° and 90°); n = 8 for alkaline-treated and densified samples per fiber angle) with a width of 10 mm and length of 100 mm were prepared and equipped with 1.4 mm thick beech veneer pads in the gripping area in order to avoid damage during tensile testing (Figure 1f). Tensile testing was carried out with a universal testing machine (Zwick-Roell 100 kN, ZwickRoell, Ulm, Germany) equipped with a 5 kN load cell (ZwickRoell, Ulm, Germany) and clip-on displacement sensors (ZwickRoell, Ulm, Germany). The initial clamp distance was 40 mm and testing was carried out at a displacement rate of 1 mm min^−1^ until failure. The initial distance of the contact extensimeter was set to 20 mm. The MOE was calculated based on the slope between 10% and 40% of F_max_. The full range of misalignment angles from parallel to the fiber axis (0°) to perpendicular to the fiber axis (90°) was tested in intervals of 10° for partially delignified and densified wood. Reference samples were exclusively tested at angles of 0° and 90°. This reduction was justified with the fact that the mechanical anisotropy of solid wood is well known and described in many widely available textbooks [11,22]. The range of tensile strength values for fiber misalignment from 10 to 80° was then calculated by means of the Tsai–Hill failure criterion:

(6) $$\frac{1}{\sigma_{x}^{2}}=\frac{cos^{4}\theta }{X^{2}}+\left(\frac{1}{S^{2}}-\frac{1}{X^{2}}\right)sin^{2}\theta cos^{2}\theta +\frac{sin^{4}\theta }{Y^{2}}$$

where $\sigma_{x}$ is the calculated tensile strength for specimens at a certain misalignment angle, θ is the angle of misalignment, *X* and *Y* are the tensile strengths parallel and perpendicular to the fiber axis, respectively, and *S* is the shear strength [23]. Shear strength of black spruce, 8.5 MPa, was taken from literature and was used for calculation [24]. The MOE values for fiber misalignment from 10 to 80° was calculated using linear orthotropic elasticity theory:

(7) $$E_{\theta }={\left[\frac{cos^{4}\theta }{E_{1}}+\left(\frac{1}{G_{12}}-\frac{2\nu_{12}}{E_{1}}\right)cos^{2}\theta sin^{2}\theta +\frac{sin^{4}\theta }{E_{2}}\right]}^{-1}$$

where $E_{\theta }$ is the calculated tensile modulus for specimens at a certain misalignment angle, θ is the angle of misalignment, $E_{1}$ and $E_{2}$ are the MOE parallel and perpendicular to the fiber axis, respectively, $G_{12}$ is the shear modulus, and *ν*_12_ is the Poisson’s ratio [25]. The shear modulus of 0.72 GPa represents a mean value based on literature [11]. The Poisson’s ratio of wood is assumed to be approximately 0.3.

For light microscopy (Axioplan 2 Imaging, Carl Zeiss GmbH, Vienna, Austria), specimens were embedded in epoxy resin (Agar Low Viscosity Resin Kit, Agar Scientific Ltd., Stansted, UK), sectioned using a diamond knife (trim 45, Diatome Ltd., Nidau, Switzerland), and observed in transmitted and/or incident light modes.

Tensile strength and MOE of partially delignified and densified samples, tested at different fiber misalignment angles, were compared statistically with their native equivalents using the statistical software SPSS (IBM SPSS Statistics, version 24, IBM Corporation, New York, NY, USA). Welch’s *t*-test was conducted comparing samples tested parallel (0°) and perpendicular (90°) to the fiber axis. One-sample *t*-test was conducted comparing tensile properties of partially delignified and densified samples tested at fiber misalignment angles from 10 to 80° with values of reference samples calculated by means of Equations (6) and (7). A 5% α-level of significance was set for both tests. The resulting *p*-value indicated the statistical significance, with *p* > 0.05 referring to no statistical significance and *p* ≤ 0.05 referring to significant differences.

## 3. Results and Discussion

The alkaline treatment described above resulted in an average loss of wood mass of 22.3%. Assuming that nearly no cellulose was degraded during the chemical treatment, it can be suspected that the mass loss resulted from the degradation of Klason lignin (6.79%) and hemicelluloses (15.51%). As a consequence, the relative amount of cellulose increased from approximately 44% in untreated wood to 56% in alkaline-treated wood. Densification resulted in specimen thickness reduction from 1.47 ± 0.02 to 0.42 ± 0.03 mm. Correspondingly, an increase in oven-dry density from 0.36 ± 0.01 g cm^−3^ for the untreated reference to 1.09 ± 0.04 g cm^−3^ for partially delignified and densified specimens was measured.

**Figure 2:**
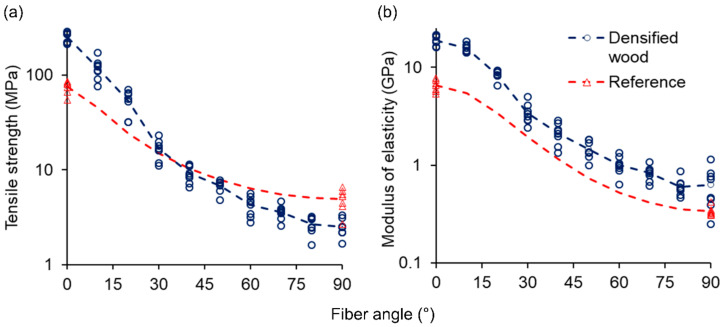
Mechanical characterization of partially delignified and densified wood at different fiber angles compared to untreated reference wood by tensile tests: (**a**) Strength; (**b**) Modulus of elasticity (log-scaling was chosen for improving the visualization of differences at high fiber angles). (n = 7 for reference samples with a misalignment angle of 0 or 90°; n = 7 for alkaline-treated and densified samples with a fiber misalignment angle of 0 or 20°; n = 8 for alkaline-treated and densified samples with a fiber misalignment angle of 10° or 30–90°).

Figure 2 shows the results of tensile tests with untreated and partially delignified and densified spruce veneers. Red triangle marks indicate strength and MOE values of untreated specimens tested parallel and perpendicular to the fiber axis. The range of strength and MOE values for misalignment angles from 10 to 80°, indicated by the red dashed lines, were subsequently calculated by applying Equations (6) and (7), respectively. Blue circle marks indicate strength and MOE values of partially delignified and densified specimens. Means are connected by the blue dashed lines. Based on these charts, distinct differences with regard to the anisotropy of strength and MOE can be revealed for treated samples. Untreated specimens exhibit an average tensile strength of 75.12 ± 11.03 MPa along the fiber direction, which is 15 times higher than the mean strength of samples tested perpendicular to the fiber axis, 4.95 ± 1.30 MPa. The MOE shows similar reduction patterns. When tested along the fiber axis, an average MOE of 6.52 ± 0.90 GPa was measured; compared with the mean value of specimens tested across the fiber direction, 0.34 ± 0.04 GPa, a reduction factor of 19 was observed. This agrees well with the reduction factors of tensile strength and MOE of wood from literature. It is well known that defect-free wood exhibits strong anisotropy of physical properties due to its distinct fibrous structure. In terms of mechanics, the strength and MOE of wood parallel to the fiber direction exceed transverse mechanical properties by a factor of 10 to 20 [11,22]. As expected based on results from the literature [16,17,18,19], the partial removal of the amorphous wood polymers (lignin and hemicelluloses) combined with a subsequent densification step led to significant improvements in tensile strength (Welch’s *t*-test, *p* < 0.05) and stiffness (Welch’s *t*-test, *p* < 0.05) parallel to the fiber direction. As shown in Figure 2, the tensile strength and MOE parallel to the fiber axis exhibit mean values of 253.21 ± 31.74 MPa and 18.86 ± 2.21 GPa, respectively. Nevertheless, similar to untreated specimens, the strength and MOE of alkaline-treated and densified veneers declined with increasing fiber misalignment angle. Mean tensile strength perpendicular to the fiber direction is reduced by a factor of 102 to 2.49 ± 0.52 MPa and is therefore even significantly lower (Welch’s *t*-test, *p* < 0.05) when compared to its untreated equivalent. MOE is reduced by a factor of 30 when alkaline-treated and densified specimens are tested at a fiber misalignment angle of 90° and exhibit a mean value of 0.63 ± 0.29 GPa. However, contrary to the tensile strength, a significant improvement (Welch’s *t*-test, *p* < 0.05) can still be observed when compared to its untreated equivalent. Interestingly, while alkaline-treated and densified samples exhibit significant improvements in MOE (Welch’s *t*-test, *p* < 0.05; one-sample *t*-test, *p* < 0.05) throughout the whole range of fiber angles, a different pattern is exhibited for the strength. Significant improvements (Welch’s *t*-test, *p* < 0.05; one-sample *t*-test, *p* < 0.05) in tensile strength can be obtained by the combination of alkaline treatment and densification until a fiber angle reaches ≤20°. In the fiber angle range from 30 to 50°, no significant differences (one-sample *t*-test, *p* > 0.05) in strength can be observed between treated and untreated specimens. When specimens are tested at fiber angles ≥60°, alkaline-treated and densified samples are even significantly inferior (Welch’s *t*-test, *p* < 0.05; one-sample *t*-test, *p* < 0.05) when compared to untreated reference.

**Figure 3:**
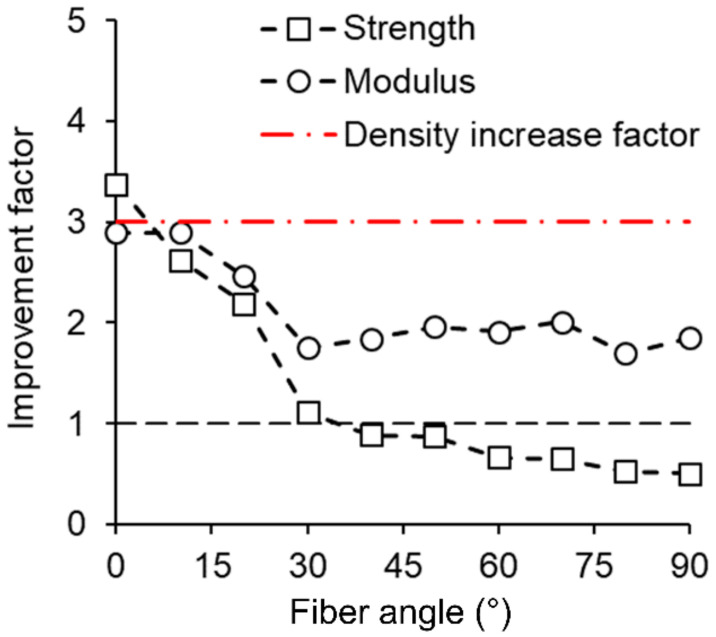
Improvement factor of the mean tensile strength and mean modulus of elasticity of partially delignified and densified wood at different fiber angles compared to untreated reference wood.

Figure 3 displays the improvement factors of alkaline-treated and densified specimens compared to reference at different fiber angles. Again, it can be clearly seen that both the strength and MOE rose significantly when tested parallel to the fiber axis and exhibited improvement factors of 3.37 and 2.89, respectively. These results are of high interest since the mechanical performance of defect-free wood correlates well with its density [26]. Comparing the improvement factor of strength and MOE tested parallel to the fiber axis with the increase factor of density, indicated by the red dash-dot line in Figure 3, a strength increase beyond the mere scaling of densification can be observed, while the MOE increased slightly less than the density. Recent literature investigating the mechanical properties in fiber direction of partially/completely delignified and densified wood obtained similar trends in strength improvements [16,17,18,19]. The concrete reason for the improvement beyond the increase in density is not yet fully understood, but three main reasons are suggested by the authors. Firstly, since cellulose is per se the strongest and stiffest component in wood, a higher cellulose content is suggested to result in better mechanical properties. Since the delignification processes described in the literature are mostly degrading hemicelluloses and lignin, an increase of the relative cellulose content is assumed, and thus an increase in mechanical performance may be expected [18]. Secondly, it was suggested that the extraction of lignin enables additional hydrogen bond formation during densification, which in turn benefits mechanical performance [19,27]. Finally, it is proposed that the removal of lignin results in a reduction of the rigidity of the cell wall [17]. When wood is then compressed transversely, a mechanical interlocking of neighboring cells at the micro- and nanoscales appears [28]. Probably the combination of the occurring alterations in wood during treatments leads to such improvements. Nevertheless, as revealed by the results shown in Figure 3, the improvement factor in strength beyond pure scaling in densification only holds true for specimens tested at a 0° fiber angle. Already when tests were carried out at 10° off-axis, the line dropped beneath the density increase factor of 3, and when tested at misalignment angles ≥30°, tensile strength was only similar or even worse for partially delignified and densified specimens when compared to the untreated reference. Comparing the improvement factors of the MOE in Figure 3, a distinct negative effect of increasing misalignment angles can be observed up to an offset angle of 30°, whereas the increase at off-axis angles >30° does not influence the improvement factor, and it leveled off to 1.8–2. Since literature predicts an essentially linear scaling of strength and stiffness with the density of wood, as is common for porous materials [29], an additional factor must come into play, causing the drastic reduction in tensile performance at higher misalignment angles observed in the present study.

**Figure 4:**
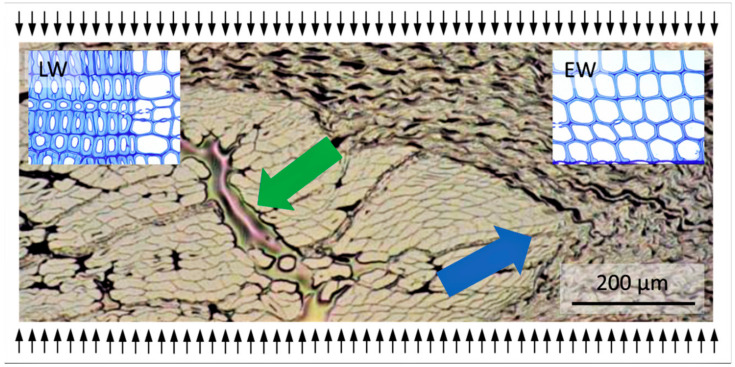
Light microscopy of partially delignified and densified wood showing latewood buckling (blue arrow) and microscopic internal cracks (green arrow) due to compression in the tangential direction (upper and bottom arrows). The inserts show representative examples for the appearance of untreated latewood (LW) and earlywood (EW).

Light microscopy of partially delignified and densified specimens quickly revealed the potential cause for the discrepancy observed (Figure 4 green arrow). Wood, as a natural material, grows inhomogeneously, with structural heterogeneity at all levels of magnification. At the level of annual growth rings, spruce wood tested in the present study showed significant differences in cell dimensions and wall thickness between latewood (LW), which is formed towards the end of the growing season, and earlywood (EW), which is formed in spring and summer (Figure 4 inserts). Upon densification, the cell walls progressively buckle and fold, until microscopic porosity is reduced to a minimum, correlating with the pressure applied [30]. Black upper and bottom arrows in Figure 4 indicate the direction of compression. It is assumed that due to structural irregularities and greatly differing densities of LW and EW, shear deformations arose during densification, which may have led to rupture of the wood structure at the interface between adjacent cells, as exemplified by the green arrow in Figure 4. This hypothesis is strengthened by the failure behavior of wood during compression found in various sources in the literature [31,32,33]. Tabarsa and Chui [31] characterized the deformation pattern of white spruce during tangential compression at a microscopic scale. It was observed that latewood tended to buckle into the earlywood regions when compressed beyond the yield point. This buckling behavior for softwood species was also noticed by Bodig [32]. Similar to what was found in the literature, a comparable buckling behavior of latewood regions was also observed in the presented microscopic images, indicated by the blue arrow in Figure 4. In a study on the characterization of the perpendicular to fiber axis compression behavior in wood construction, buckling in the direction of annual ring curvature with corresponding shear along the LW-EW interface was observed, leading to damages in the wood structure [33]. This confirms the hypothesis of an occurring separation of adjacent cells at their interfaces due to shear deformation during densification.

As revealed by data shown in Figure 2, the microcracks negatively affect both the tensile strength and the MOE in off-axis load situations. Since the MOE represents a weighted average of local material properties and is measured at low strain, it is only partially affected by the presence of microcracks. Contrarily, tensile strength is governed by the strength of the weakest link, and therefore the presence of even a small number of microcracks has a strongly negative effect on the measured tensile strength, particularly at high misalignment angles.

## 4. Conclusions

The mechanical anisotropy of partially delignified and densified wood could be demonstrated by means of tensile tests which were conducted for the whole range of misalignment angles from parallel to perpendicular to the fiber axis in intervals of 10°.

Partial delignification and densification result in a significant improvement of tensile properties parallel to the direction of wood fibers. While the MOE of treated samples exhibits significant improvement throughout the full range of misalignment angles, off-axis tensile strength exhibits a much steeper decrease, and even a significant reduction below reference values is observed at misalignment angles ≥60°. The increase in mechanical performance similar or even beyond pure scaling in densification, which is the main argument for a prior partial delignification step, only holds true if specimens are tested at 0°. Already when specimens are tested at fiber angles ≥10°, improvement in mechanical performance drops beneath the density increase factor.

It is concluded that microcracks induced by shear stress during densification are the cause of the observed reduction in off-axis performance. The possibility of damages induced by densification, which may be critical in specific load situations, should be considered in future studies with densified wood.
